# Hydration Care After Stroke: A Systematic Review of International Clinical Practice Guidelines

**DOI:** 10.3390/nu18111672

**Published:** 2026-05-23

**Authors:** Colette Miller, Elizabeth Boaden, Alison S. R. Mcloughlin, Caroline L. Watkins, Stephanie P. Jones

**Affiliations:** 1 Stroke Research Team, School of Nursing & Midwifery, University of Lancashire, Preston PR1 2HE, UK; eboaden1@lancashire.ac.uk (E.B.); clwatkins@lancashire.ac.uk (C.L.W.); sjones10@lancashire.ac.uk (S.P.J.); 2 East Lancashire Hospitals NHS Trust, Blackburn BB2 3HH, UK; alison.mcloughlin@elht.nhs.uk

**Keywords:** clinical practice guidelines, stroke, hydration, dehydration, assessment, treatment

## Abstract

**Background/Objectives:** Hydration status at the time of stroke has been identified as a predictor of both vital and functional prognosis. Many studies have demonstrated that dehydration is associated with poorer outcomes, yet the prevalence of dehydration in those affected by stroke remains high. In this review, we systematically identify, appraise and summarise international clinical practice recommendations regarding hydration care after stroke. **Methods:** International clinical practice guidelines, published since 2009, were identified through a combination of searches of four online databases, searching of relevant websites and guidelines repositories, and citation tracking. Independent screening and data extraction were followed by quality appraisal using the AGREE II tool, and qualitative content analysis underpinned by *a priori* defined categories. **Results:** Thirteen clinical practice guidelines were included, from which 35 eligible recommendations were identified. Only seven (54%) guidelines were rated as high-quality (adequately addressing at least three AGREE II domains including “Rigour of development”). The majority of the 35 recommendations were intended for application to all stroke patients (23, 66%). Specific sub-populations, for whom hydration care was emphasised included people with dysphagia (8, 23%), immobile (2, 6%) and catheterised patients (1,3%), and those with cerebral oedema (1, 3%). Hydration care was most often discussed in the context of the avoidance and/or management of post-stroke complications, with only 8 (23%) recommendations specifically discussing hydration care alone. Of those eight recommendations, 3 (38%) suggested all stroke patients should have their hydration assessed, and 5 (62%) proposed methods of hydration management. **Conclusions:** The review demonstrates that international stroke guidelines recognise the importance of hydration care, although almost half of the guidelines are low to moderate quality and consider hydration in the context of post-stroke complications. Whilst hydration care, routine assessment and management of hydration status, is broadly endorsed, methods remain poorly defined. Further high-quality evidence is needed to support the development of standardised, evidence-based guidelines. Future guidelines should address the timing and methods of assessment, including the establishment of diagnostic thresholds to inform the interpretation of haematological results and subsequent treatment decisions.

## 1. Introduction

Water is an essential nutrient, constituting around two-thirds of the human body, and a necessary component of many essential physiological processes [1]. It is vital for life, making up cells, tissues and organs, yet the human body lacks the capacity to produce it, meaning it must be ingested to meet metabolic requirements [2]. Adequate hydration is imperative for general health and wellbeing; consequently, it is recommended that water be consumed daily to promote optimal physiological functions such as digestion, metabolism and thermoregulation [3]. The primary goal of oral hydration is to prevent dehydration, even mild cases of which can result in mood changes, constipation and kidney stones; however, hydration is particularly important at times of illness, when the body’s requirement for nutrients increases to aid repair and recovery [4]. Mild dehydration is believed to play a role in the development of various morbidities, whilst good hydration has been associated with reductions in hypertension, fatal coronary heart disease and stroke [5].

Stroke is the second leading cause of death and third leading cause of disability globally, with over 12.2 million new strokes occurring each year [6]. Around half of acute stroke patients will become dehydrated during their hospital stay [7,8]. Dehydrated stroke patients are four times more likely to demonstrate early clinical worsening when compared with their adequately hydrated counterparts, and dehydration is independently associated with longer length of stay and greater costs [9,10]. Patients with dehydration at any single point during their hospital stay are significantly more likely to die, or to be dependent upon discharge [7,11,12,13,14,15].

Conversely, correction of dehydration may prevent the occurrence of stroke in evolution [16,17], reduce the development of infections and venous thromboembolism [18,19,20], and improve stroke patient outcomes [21]. Of note, the benefits of effective hydration care have been exhibited not only in dehydrated stroke patients, but also in those initially presenting without dehydration [22]. Furthermore, whilst recent clinical advances provide tremendous opportunities for treatment, the majority of stroke patients are not eligible for hyper-acute medical and/or surgical interventions, but can still benefit from fundamental aspects of organised in-patient stroke unit care [23]. For example, the use of thrombolytic therapy does not eliminate the need for skilled care, including physiological management such as the close monitoring of hydration status, in patients with stroke [24].

Despite the recognised potential of hydration care to improve patient outcomes, quality improvement initiatives have yielded mixed results [25,26], and the prevalence of dehydration in the stroke population remains high (29–70%) [27]. Monitoring and assessment of hydration status may be low on the hierarchy of priorities for healthcare practitioners when faced with the medical emergency represented by stroke. Consequently, hydration care tends to be intermittent, rather than routine, occurring in response to other clinical issues [26,27,28]. Further challenges, expressed by healthcare practitioners, include a perceived lack of training and evidence-based protocols to guide and standardise practice regarding hydration care [28]. Evidence supporting these perceptions emerged from a survey of hydration care in Australia and the United Kingdom (UK) which found that practice varied, and the assessment of hydration status was not routinely informed by structured screening tools [29].

Variations in practice may be explained, in part, by the equivocal evidence-base available to inform hydration care. A Cochrane review, exploring methods to aid the identification of dehydration in older people, found little evidence that any one symptom, sign or test has diagnostic utility [30]. Whilst a further Cochrane review found no evidence to guide the correct volume, duration, type~ or mode of fluid delivery in acute stroke care [31]. This uncertainty is further compounded by considerable variation in the definitions of dehydration, diagnostic tests and thresholds used in published research [27].

Hydration care after stroke presents an important clinical problem, and opportunity, with no apparent consensus regarding the optimum methods of assessment and management. In such circumstances, healthcare practitioners customarily rely on clinical practice guidelines (CPGs)—statements, often informed by systematic review, that include recommendations intended to optimise patient care [32]. However, the potential of such CPGs to enhance practice in relation to hydration care is dependent on the availability and quality of the underpinning evidence. Additionally, the recent proliferation of CPGs has raised questions concerning their quality, necessitating robust appraisal prior to clinical application [33].

This review sought to identify, appraise and summarise recommendations in international CPGs regarding hydration care, namely the assessment and management of hydration status, and any subsequent diagnosis and treatment of dehydration, in the acute stroke setting.

## 2. Materials and Methods

### 2.1. Review Protocol

The review was conducted according to the method proposed by Johnston and colleagues [34] and is reported in accordance with the Preferred Reporting Items for Systematic Review and Meta-Analysis (PRISMA) guidance [35]. A PRISMA checklist is provided in Supplementary Materials S1. A protocol was prospectively registered on the international prospective register of systematic reviews PROSPERO: CRD42020217931.

### 2.2. Eligibility Criteria

The ‘Population, Intervention, Comparator, Attributes of CPG, and Recommendations’ (PICAR) Framework informed question development, inclusion/exclusion criteria, and subsequent screening [34,36]. The PICAR statement can be seen in Table 1. CPGs were eligible for inclusion if they:

- Included recommendations specifically pertaining to hydration care—the assessment and management of hydration status, and any subsequent diagnosis and treatment of dehydration—in the acute stroke setting;
- Were endorsed by a national and/or international organisation (e.g., government, professional, third sector);
- Were published from January 2009 (to ensure only the most up to date CPGs were included);
- Were available in English.

Identified CPGs were excluded if they:

- Related only to specific neuro (surgical) techniques for the treatment of stroke;
- Focused exclusively on care outside the acute setting (e.g., primary prevention, rehabilitation, and community).

### 2.3. Search Strategy and Guideline Selection

Boolean search strategies using a combination of Medical Subject Headings (MeSH) and free-text keywords were constructed to retrieve the relevant literature published between January 2009 and the 16th of February 2026. The online bibliographic databases MEDLINE, Health Management Information Consortium (HMIC), Embase, and CINAHL were systematically searched using database specific controlled vocabulary, and filters to restrict by publication date. The Ovid platform was utilised to access three databases, and CINAHL was accessed via the EBSCO platform. The full search strategy and prompts used for each database are provided in Supplementary Materials S2.

CPGs can be challenging to identify through bibliographic database searches alone, as they are frequently produced by professional organisations or governmental bodies and are not consistently indexed within standard databases. Consequently, reliance solely on database searching may result in the omission of the relevant and important literature [37]. To overcome this barrier to effective data retrieval, websites and guideline repositories, including but not limited to, the Guidelines International Network (https://www.g-i-n.net/ (accessed on 7 October 2025)), The American Academy of Neurology (https://www.aan.com/ (accessed on 7 October 2025)), The European Stroke Organisation (https://eso-stroke.org/eso-guideline-directory/ (accessed on 7 October 2025)), and Google/Google Scholar were systematically searched to identify additional CPGs. Two authors (CM and AM) independently screened titles and abstracts, followed by full-text assessment of potentially eligible records. Disagreements were resolved through discussion with the review group. Forward and backward citation tracking of included guidelines was conducted to identify further eligible publications. The final list of included guidelines was reviewed by all authors to ensure that no relevant CPGs known to the research team were omitted.

### 2.4. Data Extraction and Quality Appraisal

The lead author was responsible for data extraction using a bespoke proforma, which was piloted with three CPGs and revised before being finalised. The following information was extracted: authors; organisation; year of publication; geographical remit; development approach; methods of evidence and quality assessment; recommendation development approach; funding and disclosures; and all content regarding hydration care in acute stroke. Data was also collected regarding the *a priori* defined questions (PICAR—“(key) Content”) which the review sought to answer and can be seen in Table 1. Where necessary, Supplementary Material was retrieved to inform both data extraction and quality appraisal. The completeness and accuracy of data extraction was checked by a second reviewer (AM).

The quality of included CPGs was assessed using the Appraisal of Guidelines for Research Evaluation Tool (AGREE II) [38,39]. Four members of the review team (CM, AM, EB and SJ, with clinical expertise in stroke and/or applied stroke research) independently assessed each guideline to inform the development of a quality score for each of the six quality domains in line with the AGREE formula [39]. Agreement was assessed for each domain item, and scores > 1.5 SD from the mean item score were collectively reviewed and discussed until agreement was reached [40]. A score of ≥60% was sufficient to demonstrate a domain had been adequately addressed [40,41,42,43,44]. Overall quality of guidelines was assessed with the application of a standardised scoring rubric, described previously by Johnston and colleagues [40]. Guidelines assessed as ‘high’ quality adequately addressed at least three of the six domains (including domain 3 ‘Rigour of development’); ‘moderate’ quality were defined as addressing at least three of six domains (not including domain 3), or addressing at least two domains and achieving a score of at least ≥50% in domain 3; ‘low’ quality judgements were assigned to guidelines which did not meet any of the above criteria.

### 2.5. Data Analysis and Synthesis

Recommendations and informal text explicitly referring to hydration care in acute stroke were collated in matrices to facilitate comparison of content and categorisation of the data [40,45]. The “(key) Content” item of the PICAR formed the basis of an initial guide to inform directed content analysis—the reduction of data into defined categories to facilitate descriptive knowledge and understanding [46]. The five *a priori* defined categories were: patient population(s); assessment and investigation; diagnosis; management and treatment; and patient interventions. Following categorisation, a structured narrative synthesis was undertaken.

## 3. Results

### 3.1. Literature Search

**Figure 1:**
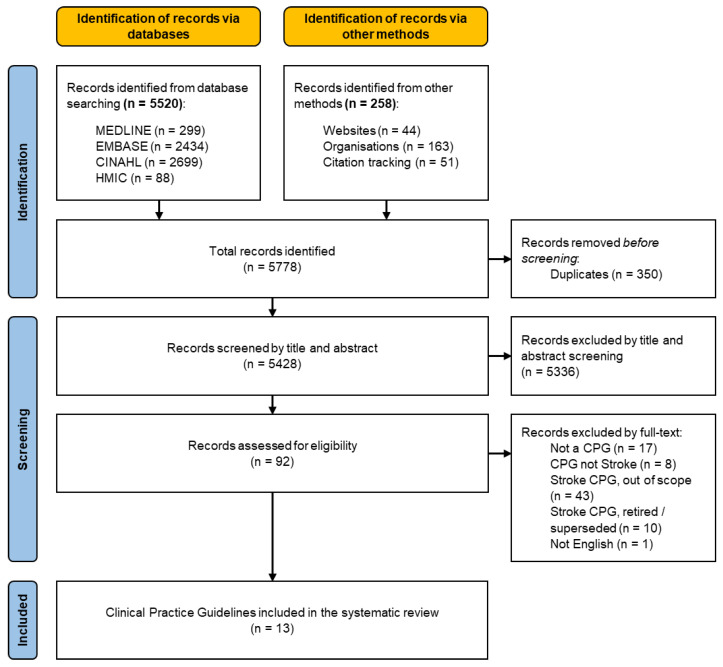
PRISMA flow diagram of study selection.

The results of systematic searching, screening and selection are summarised in a PRISMA flow diagram (Figure 1). A total of 5778 records were identified from searching (described in Section 2.2). After de-duplication, title and abstract, and full-text screening, thirteen international CPGs were included in the review.

### 3.2. Characteristics of Included Guidelines

The characteristics of the 13 included CPGs are shown in Table 2. One CPG [47] was published in 2009, four between 2010 and 2019 [48,49,50,51] and eight from 2020 to 2026 [52,53,54,55,56,57,58,59].

Across the 13 guidelines, there was variation in both the quality of the evidence underpinning recommendations and the approaches used by guideline developers to assess it (see Table 2 and Supplementary Materials S3). Five CPGs explicitly reported the use of established evidence grading frameworks such as the Grading of Recommendations Assessment, Development and Evaluation (GRADE) approach [48,49,50,57,59]. A further five used frameworks adapted from established frameworks, modified to local context where appropriate, including GRADE and the ‘American Heart and Stroke Association’s Class of Recommendation/Level of Evidence’ methodology [47,52,53,54,56]. Two CPGs did not identify their evidence assessment by name, and one reported that ‘expert consensus’ and ‘the best available evidence’ were prioritised over formal grading assessments [48,51,57]. Whilst nine of thirteen CPGs attempted to grade the strength of recommendations and quality of underpinning evidence, there was substantial heterogeneity in the frameworks applied. Where formal systems were applied, recommendations were often underpinned by low to moderate levels of evidence, or consensus based and good clinical practice recommendations [48,51,53,57,59].

### 3.3. Quality of Included Guidelines

Table 3 shows the AGREE II scores of included CPGs, with 39 (50%) domains scoring ≥ 60%. Domain one, ‘Scope and Purpose’, was the highest scoring domain, with 10 (77%) CPGs scoring ≥ 60% (range 68–100%). Domain four, ‘Clarity of Presentation’, followed with 8 (62%) CPGs being scored as adequately addressing the domain. The key domain which informed overall quality assessment, domain three ‘Rigour of Development’, was scored ≥60% in 7 (54%) of CPGs. The lowest scoring domains were two ‘Stakeholder Involvement’ and five ‘Applicability’, with only 4 (31%) CPGs scoring ≥ 60% in either domain. The median score for, ‘Applicability’, was 13%; however, three guidelines were scored zero, and four over 60%. Three CPGs scored ≥ 95% in a single domain.

More than half (N = 7, 54%) [52,53,54,55,57,58,59] of included CPGs adequately addressed at least three of the six domains, including domain three, and were judged to be of high overall quality. One CPG [57] was judged to be of moderate quality, scoring > 70% in three domains excluding number three, with the remaining five CPGs [47,49,51,52,55] being judged as low overall quality. None of the CPGs judged as being low in overall quality scored above 50% in domain three (range 2–44%).

### 3.4. Recommendations

A total of 35 clinical practice guideline recommendations (CPGRs) relating to hydration care were extracted from the thirteen CPGs and included in the analysis (a full list of which can be seen in Supplementary Materials S3). The number of CPGRs extracted from each CPG ranged from one to six. Areas of stroke care from which hydration care CPGRs were extracted, as categorised in the source CPGs, included: stroke unit organisation (1, 3%); pre-hospital (1, 3%); emergency department (2, 6%); assessment (3, 9%); management (4, 11%); nutrition (including with hydration, or with dysphagia) (8, 23%); and specific complications including deep vein thrombosis (DVT) (6, 17%), dysphagia (9, 26%) and intracranial pressure (1, 3%).

The data mapped to three of the five *a priori* defined PICAR categories. No data informed the clinical diagnosis of dehydration, nor were definitions provided to guide the classification of normal or adequate hydration status. None of the CPGs included content regarding patient-led interventions. Specific sub-populations with additional complications were frequently referenced in relation to hydration care, resulting in the emergence of an additional category “Post-stroke Complications”. A structured narrative summary of categorical findings is presented below.

#### 3.4.1. Patient Population(s)

The majority of the 35 CPGRs were intended for application to all stroke patients (23, 66%). Specific sub-populations, for whom hydration care was emphasised included people with dysphagia (8, 23%), immobile (2, 6%) and catheterised patients (1, 3%), and those with cerebral oedema (1, 3%).

Hydration care was most often discussed in the context of the avoidance and/or management of common post-stroke complications, with only 8 (23%) CPGRs from 7 countries, specifically discussing hydration care alone [48,49,50,52,53,57,58]. Of those, 3 (38%) suggested all stroke patients should have their hydration assessed [48,54,58], and 5 (62%) proposed methods of management and/or treatment suitable for all patients [48,49,50,53,58]—the details of which are reported in the relevant sections below.

#### 3.4.2. Assessment and Investigation

Of the included CPGs, 9 (69%) provided specific recommendations, or informal text, highlighting the importance of hydration care [49,50,51,52,53,55,56,57,58]. Terms used in CPGRs to describe such care included: assess, evaluate, investigate, monitor, manage and observe. Specific timescales for the completion of assessment were provided by 3 CPGRs with timings ranging from 4 to 24 h [50,58]. The third CPGR providing information regarding the timing of assessment, was of notable asthe only CPGR to require that clinical evaluation of hydration status be carried out during pre-hospital transportation [49]. Two CPGRs suggested that regular assessment, monitoring and management should occur throughout admission, presumably commencing on admission, though the latter recommended this only in the case of dysphagia or severe stroke [48,57]. The methods by which hydration status should be assessed varied, including: a ‘standardised approach’; validated screening tools; haematological investigation; urea and electrolytes; and monitoring of fluid balance/urine output [50,54,57,58]. No CPGRs specified which healthcare practitioners should take responsibility for the implementation of recommendations relating to hydration care alone, and no detail was provided regarding specific diagnostic thresholds for the interpretation of haematological results. One CPGR stated that all acute stroke services should have management protocols for hydration and nutrition care [58].

#### 3.4.3. Management and Treatment

The overarching rationale for hydration care was the maintenance of ‘adequate’, ‘good’, ‘normal’ or ‘optimal’ hydration status. Details regarding the timing of management and treatment interventions were sparse. Recommendations stated that patients should be ‘…reviewed regularly and managed’ [], and fluid support/infusion should be given where required/when necessary [48,51]. The most commonly cited fluid replacement option was IV fluids, with 7 (20%) CPGRs discussing this approach [49,50,51,53,54,56,58], all of which stated that isotonic (crystalloid) solution should be utilised. Only the Qatar CPGR provided guidance on ‘what not to do’, stating that hypotonic fluids should not be used with those affected by stroke [56]. One CPGR provided detail to guide the volume of IV fluid administration, 100mL/hour, and highlighted the need for consideration of an individual’s baseline hydration status and co-morbidities prior to fluid prescription [49]. Two CPGRs specified the duration of IV fluid therapy. A South African CPGR stated that all patients should receive IV fluids for the first 24 h [57], and in India, those with altered sensorium were recommended to receive only IV fluids for at least 2–3 days [52]. Two CPGRs included information regarding restriction of oral hydration [54,55]. In Malaysia, informal text stated that patients be “kept nil-by-mouth” and “put on an IV drip”, though this was not included in formal recommendations and, in Pakistan, the CPGR stated that free fluids should be “avoided”. This approach was mirrored in other CPGs when considering patients with specific complications which are presented in Section 3.4.4. Good positioning was also highlighted in one CPG as a mechanism to avoid compromising hydration and nutrition, though again this advice did not meet the threshold for inclusion in formal recommendations [58].

Dehydration was explicitly mentioned in 7 (20%) CPGRs from four (31%) CPGs [48,49,52,56]. Management options included ensuring that the risk of dehydration is included in history taking and observing patients for the development of ‘common early complications including dehydration’ [52,56]. Methods for the treatment of dehydration were detailed in Australian and Brazilian CPGRs, with both recommending crystalloid (0.9% normal saline) as the first option to prevent or treat dehydration [48,49].

#### 3.4.4. Post-Stroke Complications

Hydration care in relation to the assessment, prevention and treatment of deep vein thrombosis (DVT) was the focus of 6 (17%) CPGRs, from six countries [47,50,51,52,54,57]. Dehydration was identified as a risk factor to be aware of when assessing patients for the risk of developing DVT. For all stroke patients, interventions to prevent the occurrence of DVT included, “good” and “optimal” hydration, or “early” rehydration. For those with limited mobility, conventional treatment/routine care was described as a combination of aspirin and “fluid therapy” or “hydration”.

Of the 35 included CPGRs, 14 (40%) from nine countries were concerned with dysphagia. Four related to screening for swallow impairment, suggesting this be completed before oral intake (nutrition, hydration and medication) commences [50,51,52]. A Canadian CPGR specified that this should coincide with assessment of hydration status and take place ‘as early as possible, ideally within 24 h of admission, using validated screening tools’ [50]. They further stated that abnormal swallow screen results should ‘trigger prompt referral to speech-language pathologist, occupational therapist, dietitian and/or other trained dysphagia clinicians for more detailed assessment and management’ of hydration status amongst other clinical issues. Two CPGRs recommended for all patients that, until a safe swallow has been confirmed, hydration be assessed and managed to achieve adequate levels of hydration, with early or immediate consideration of non-oral routes [48,58]. For those unable to meet their fluid needs orally due to confirmed dysphagia, CPGRs to guide management varied slightly in their stance with the Qatar CPGRs suggesting patients ‘may benefit from IV fluids to maintain hydration’ [56] and the Indian and Japanese GPGRs recommending that IV fluids be prescribed with avoidance of oral intake [52,53]. Following at least 2–3 days of IV fluid (normal or dextrose) the Indian CPGR stated that nasogastric (NG) tube feeding be initiated, which was mirrored by the Canadian equivalent requiring NG be considered ‘as early as possible’, but within three days of admission [50,52]. This was the only CPGR to stipulate that consideration of enteral routes be undertaken in consultation with the patient, family or substitute decision-maker [50]. Other instances where dysphagia care and hydration care intersected included, reviewing patient tolerance of modified diets to avoid dehydration [48], regular monitoring of fluid balance and electrolytes [57] and urgent review of those with dysphagia identified as being dehydrated [48].

Monitoring of fluid balance was also deemed important in the context of other common post-stroke complications. Strict monitoring of fluid status was recommended as part of routine monitoring of vital signs for those with indwelling urethral catheters [50], and strict intake-output charts were recommended to avoid dehydration in those with cerebral oedema [52].

## 4. Discussion

This is the first systematic review of international clinical practice guidelines to identify, appraise and summarise the recommendations on hydration care in acute stroke. Across the reviewed guidelines, consensus exists regarding the importance of adequate hydration and its potential to improve clinical outcomes, yet there is a lack of consistency and clarity on the exact methods to be utilised in effective hydration care after stroke. Although the importance of routine assessment of hydration status was acknowledged, this recognition was not consistently reflected in formal recommendations. Hydration care was most frequently discussed in terms of the avoidance of complications, or in the context of common post-stroke co-morbidities such as dysphagia, deep vein thrombosis and in one instance intracranial pressure. This hierarchy appears to be mirrored in practice, with hydration care being undertaken in response to other clinical issues, lacking standardisation in line with CPGs and remaining poor in comparison with best practice—even following quality improvement initiatives [25,26]. Further high-quality research is essential to inform the development of robust guidelines for hydration care, including accurate diagnosis and effective treatment of dehydration, in acute stroke.

Based on the AGREE II appraisal, although the quality of included CPGs varied, just over half were rated as high quality. Domain 1 (scope and purpose) and Domain 4 (clarity of presentation) were the highest scoring, consistent with recent systematic reviews of stroke CPGs regarding cryptogenic stroke and the use of thickened fluids [44,60]. ‘Applicability’ (Domain 5) and ‘Stakeholder Involvement’ (Domain 2) were the lowest scoring items of the AGREE II instrument. Applicability relates to the implementation of recommendations, including advice and/or tools to guide local adoption, information regarding the resources required, and consideration of facilitators and barriers to effective uptake [38]. Of the nine CPGs which did not adequately address ‘Applicability’, three scored zero, and the remaining five had low scores ranging from 4–18. These results are in line with similar reviews of guideline quality, with ‘Applicability’ consistently identified as an area where great improvement is possible [33]. In practice, barriers to effective implementation can arise at all stages of the healthcare pathway, from patient to provider, requiring consideration of the various opportunities for success—and failure—early in the implementation stage [61]. Whilst high quality guidelines should include information to guide implementation, they are not ‘recipe books’ and will likely have limitations in their applicability to local contexts [62]. It is, therefore, imperative that health care providers and professionals adapt and tailor guidelines to the local context within which they are applied [63]. Nevertheless, guideline developers could substantially improve the uptake and adoption of recommendations by addressing economic and resource issues facing those expected to implement them, and through the provision of frameworks to guide the evaluation of adherence to recommendations [33,38,61].

Just as ‘Applicability’ is crucial to the implementation of guideline recommendations, ‘Stakeholder Involvement’ is an important component of guideline development which aims to ensure that CPGs are patient-centred, incorporate lived-experience and identify outcomes that matter most to those potentially affected by their recommendations [64]. The nine CPGs which scored below the threshold to be judged as adequately addressing stakeholder involvement were published between 2010 and 2026, with the majority being published since 2020, suggesting that age alone does not explain the lack of stakeholder involvement and indicating space for improvement in this area of guideline development. Issues facing guideline developers in the engagement of people and communities may include resources, recruitment, and participants’ lack of familiarity with complex medical and scientific terminology [65]. Additionally, despite acknowledgement of the need for patients to be meaningfully and equitably involved in the development of guidelines, there is a lack of guidance regarding effective multi-stakeholder engagement [66]. Recent, and ongoing research, seeking to describe methods of engaging with, and to develop a checklist for when and how to involve, stakeholders in health guideline development, have the potential to enhance engagement strategies in the development of future guidelines [67,68].

In addition to involving patients in guideline development, engaging them in their care offers an opportunity to improve the quality and delivery of health services [69]. Patient-led interventions, whereby patients or carers are educated and empowered to take an active role in managing their own care, can improve treatment outcomes and patient satisfaction [70]. Indeed, health promotion and health education in hydration have been shown to be effective across a number of outcomes including reductions in falls, delirium, urinary tract infections and hospital length of stay [71]. Only one CPGR referred to shared-decision making, in consultation with the patient or their family, regarding consideration of enteral nutrition, and no CPGRs referenced patient-led interventions [50]. This may be explained by the complex nature of acute stroke care, and the significant barriers to patient engagement including reduced consciousness and cognitive deficits [72]. Nevertheless, quality improvement initiatives which sought to improve hydration in hospitalised adults, ranging from simple sticker placement on patient cups to multi-component interventions, have demonstrated promising results [73,74]. Key contributing factors to the success of such initiatives were implementation of local protocols, appropriate healthcare professional education to improve knowledge regarding hydration and multi-disciplinary team (MDT) working [71,72,74].

Whilst MDT working is crucial in all aspects of stroke care, without clarity as to the specific contributions of team members, hydration care becomes ‘everybody’s and nobody’s job’ [28]. None of the CPGRs contained information to determine which members of the MDT were best placed to take responsibility for hydration care, though the Canadian recommendations did specify which professions should be involved upon confirmation of dysphagia [50]. Owing to their close and frequent contact with patients, nurses and resident doctors have been postulated as the obvious choice to spearhead improvements in hydration care, acting as ‘champions’ to increase MDT awareness and engagement [28,71]. Regardless of which profession(s) take the lead, recent evidence suggests that all members of the MDT would benefit from the development and implementation of continuing education programs, workshops and conferences covering topics surrounding hydration care [26,28,74,75]. The enhancement of practitioner’s knowledge and skills in this area may result in more efficient and effective hydration care, including the assessment and management of hydration related complications [71,72,73,74,75].

Guidelines were particularly vague regarding a critical aspect of hydration care, the assessment of hydration status, suggesting that patient risk of dehydration should be clinically evaluated using a ‘standardised approach’ informed by validated screening tools [49,50,52,58]. The only specific clinical test recommended to inform the assessment of hydration status was the investigation of urea and electrolytes (U&Es), and no definition was provided to guide the classification of hydrated versus dehydrated status [54]. Whilst U&Es provide a broad overview of kidney function and hydration status, such biomarkers are not specific to hydration care and can be influenced by other pathologies such as cardiovascular, kidney and liver diseases, and endocrine disorders [76,77,78]. In the absence of definitive scientific evidence to guide routine clinical evaluation and treatment approaches for dehydration in acute stroke, reliance on such indirect biomarkers is common [9,79,80]. It is acknowledged that serum osmolality is the reference standard for the diagnosis of dehydration in older adults; however, measurement is invasive and may be costly, leading researchers to explore alternative approaches to identify dehydration [30,80,81]. Recent research suggests that a combination of physiological and biochemical indicators such as blood pressure, heart rate, haematocrit levels, urinary output and serum or urine osmolarity may provide a more comprehensive evaluation, but further research is needed to assess the utility of these approaches [30,80,82]. Exploration of effective combinations seems reasonable, and it would appear prudent to equip practitioners with a variety of methods for the assessment of hydration status given that physiological, rather than biochemical, parameters have been shown to be practical indicators of mild dehydration in older hospitalised adults [83]. However, the identification and validation of a single, objective measure to clinically diagnose dehydration, and inform routine monitoring of hydration status in stroke patients, remains a key priority for researchers [26].

Methods suggested for the monitoring of hydration status included U&Es, the limitations of which have been discussed previously, and fluid balance charts [52,57]. Previous research suggests that completion of fluid balance charts is the most common method of documenting the oral intake of stroke patients [29]. Fluid balance is a modifiable risk factor and safety endpoint for evaluating the effectiveness of fluid therapy, the goal of which is to achieve neutral fluid balance [84]. Despite fluid balance charts being an essential part of clinical nursing documentation, the quality of completion has been reported as inadequate, incomplete and incorrect—specifically due to human calculation errors [28,85]. Reasons proposed for poor completion include poor communication between staff and a lack of training, and quality improvement projects including education and training have been shown to remarkably improve completion [28,86]. In patients with severe ischaemic stroke, positive fluid balance (reflecting hypervolemia) in the first three days has been associated with higher risk of mortality, highlighting the complexity of hydration care [28,84]. Similarly, whilst research has focused on the effects of hypo-osmolality, Li and colleagues identified an association with both hyper- and hypo-osmolality and mortality, suggesting that regular and careful monitoring of hydration status is required to support improved outcomes in stroke care [87]. Without clear guidance detailing the specific physiological and biochemical markers of interest, including critical thresholds to signal the need for action, practitioners may be unable to effectively monitor hydration status after stroke.

Clarity and specificity in guideline recommendations, such as defining patient populations and specific interventions, is imperative to ensure that practitioners are able to interpret and implement them in a time-efficient manner [88]. Vague language in recommendations has the potential to cause confusion or lead to a variety of interpretations, adding additional layers of complexity for those expected to implement them. Terms used to describe hydration care in stroke guidelines included, ‘adequate’, ‘good’, ‘normal’ or ‘optimal’, none of which were sufficiently specified to inform decision-making, minimise errors, or reduce unwarranted variation in practice [47,49,52,58]. Where intervention was recommended, usually the provision of intravenous fluid replacement, only one recommendation included detail regarding the timing, type, volume and duration of fluid delivery—suggesting normal saline (0.9%) infusion at a rate of 100mL per hour [49]. An explanation for the lack of detail supplied in other recommendations could be that guidelines by their very nature, are required to be adaptable across a variety of clinical settings, patient populations and staff groups, and to support rather than replace clinical judgment. Perhaps guideline developers, mindful that local contextual considerations often influence implementation, have avoided the stipulation of specific hydration care interventions to allow practitioners professional judgment to inform care? [89]. It must be noted that the one guideline providing detail regarding specific treatment methods did so with the caveat that any prescription must be considered in the context of individual patient factors—thereby leaving space for local application [49]. Another confounding factor faced by guideline developers in the provision of actionable recommendations for hydration care is the evidence base from which they are generated. To be well-developed, it is accepted that guidelines should be based on the best available and most recent scientific evidence, however such evidence is lacking in this area of post-stroke hydration care, with no consensus existing to guide effective care [27].

The lack of specificity was again apparent when hydration care was discussed in the context of the avoidance of DVT after stroke [47,50,51,52,54,57]. For those able to move, early mobilisation and hydration were recommended [47,52,57], and for patients whose mobility was limited, routine care included hydration/fluid therapy [51,54]. Guidelines including this approach were based upon guidelines from the USA which, until the 2026 update, included information specifying that routine care for this population included “aspirin and hydration” [90]. The supporting text to this recommendation outlined that the definition of routine care was derived from the underpinning research upon which it was based, the Clots in Legs Or sTockings after Stroke (CLOTS) 3 trial [90]. Hydration care was not the focus of CLOTS 3, the trial protocol did not specify what hydration care should be provided, nor did the researchers collect information regarding fluid prescription during the trial period [91,92]. In the 2026 update of the American guidelines, the details of “aspirin and hydration” were removed, leaving only “routine care”, increasing the ambiguity of the recommendation [59]. Essentially, recommendations informed by this evidence contain no information to guide effective hydration care, assuming instead that local protocols will include such details.

It would appear logical to presume that care settings have protocols to guide fundamental aspects of care such as hydration. The UK guidelines recommended that acute stroke services should have management protocols for hydration and nutrition, and the informal text of guidelines for Australia and New Zealand stated that 93% of hospitals reported having locally agreed management protocols for hydration [48,58]. Despite this, research carried out in both countries found that no standard protocols existed to guide hydration care after stroke, instead practitioners relied upon assessment methods that have been shown to be poor indicators of hydration status, and initiatives to improve hydration care had limited success [26,28,29]. The research highlights the discrepancy between guidelines and practice, further confirming issues raised previously surrounding the applicability of guidelines and demonstrating that further effort is required to increase uptake and adoption of recommendations.

### Strengths and Limitations

To the best of our knowledge this review is the first published appraisal of clinical practice guideline recommendations focused on hydration care in acute stroke. This review has several strengths, including a pre-registered protocol, adherence to PRISMA guidelines and the use of the AGREE II framework to appraise the quality of included guidelines. Nevertheless, several limitations are acknowledged. Over one third of the guidelines included were published between 2009 and 2019 and consequently could not reflect the most recent evidence in this area. Methodological quality varied with several guidelines providing limited detail on systematic searching methods, underpinning evidence, and approaches to synthesis and recommendation development. Due to limited resources, it was only possible to include guidelines published in English, though efforts were made to access translated versions where possible. Similarly, whilst our best efforts were made to identify all eligible guidelines published in English, and all Supplementary Material to inform guideline appraisal and interpretation, the possibility that some may have been missed cannot be ruled out. All members of the review team were from the UK, which raises the potential for geographical bias, though our use of the validated and reliable AGREE II instrument should minimise this risk. The results of this review should therefore be considered in the context of these limitations.

## 5. Conclusions

This review examined recommendations from international clinical practice guidelines regarding hydration care in the acute stroke setting. The extracted data mapped to three of the five *a priori* defined PICAR categories. No data informed the clinical diagnosis of dehydration, nor were definitions provided to guide the classification of normal or adequate hydration status. None of the guidelines included content regarding patient-led interventions. Although it is clear guidelines acknowledge the importance of hydration care, and its potential to improve clinical outcomes, this recognition is not consistently reflected in formal recommendations. Hydration care was most often discussed in relation to the prevention of complications, or the management of common post-stroke comorbidities, which may result in reactive and variable clinical practice. Whilst recommendations regarding specific post-stroke complications or comorbidities included somewhat more detail, guidelines do not clearly articulate what should be done when and how for all patients.

The review findings highlight important implications for clinical practice, particularly the need to improve clarity and consistency in hydration care for people with stroke. Variability in assessment methods, timing and management approaches may contribute to inconsistent care delivery and potential patient risk. To address this, practitioners should consider the development and implementation of standardised hydration care protocols, as recommended in some included guidelines, beginning at admission and continuing throughout care [48,49,58]. The integration of hydration care across stroke care pathways, including coordinated responsibility across multidisciplinary clinical teams, may improve the consistency, safety, and effectiveness of hydration care in clinical practice.

The findings also reinforce the need for further research to identify an optimal pathway, and inform standardisation of care, for the investigation and management of hydration status in the acute stroke population. Whilst clinical guidelines are not a substitute for clinical judgement and must be adapted to the local context in which they are applied, the development of more robust primary research would enable future guideline iterations to draw on a stronger evidence base and develop consistent evidence-informed approaches to hydration care. Unless addressed, this gap in knowledge will continue to result in missed opportunities to advance a fundamental aspect of patient care, and to improve outcomes for those affected by stroke.

## Figures and Tables

**Table 1:** PICAR Statement, inclusion criteria for the review.

	PICAR Item Definition	PICAR Items Relevant to Screening CPG/Rs for Inclusion
**P**	*Population*, *clinical indication(s)*, *and condition(s)*	Guidelines for adults (>18 yrs) affected by stroke, who seek or receive care from healthcare practitioners in an acute hospital setting.
**I**	*Intervention(s)*	Explicit recommendations that address any strategy for the assessment, diagnosis, treatment and management of hydration status that can be applied in an acute hospital setting by healthcare practitioners.
**C**	*Comparator(s)*, *comparison(s)*, *and* *(key) content*	(key) Content of interest includes: **PATIENT POPULATIONS** Which patients should have their hydration assessed? Are there any exceptions? Are certain patient populations at higher risk? And therefore require alternative pathways? Is there a way to group the patient populations to inform practice? **ASSESSMENT/INVESTIGATION OF HYDRATION STATUS (DETECTION OF DEHYDRATION)** What actions (assessment of clinical signs, tests etc.) do the guidelines recommend? By whom should these actions be carried out? When and how often should they be actioned? Are specific diagnostic tests/measures recommended? e.g., BUN/Cr, pOsm/uOsm **DIAGNOSIS** Do the recommendations include information to guide the formal diagnosis of dehydration? If yes, what are the thresholds for diagnosis? **MANAGEMENT OF HYDRATION STATUS/TREATMENT OF DEHYDRATION** Do the guidelines recommend any "normal ranges"/thresholds for routine observations e.g., BP/HR/Bloods to determine "adequate" hydration status? Are there any variations in recommended thresholds based on patient characteristics? e.g., CHF, diabetes etc. In the event of Dehydration diagnosis, are specific interventions recommended? If so, is there guidance regarding the timing, type, dose, and duration of treatment? **PATIENT INTERVENTIONS** Are there any recommendations regarding patient/carer interventions? Behaviour change/psychosocial Educational
**A**	*Attributes of the CPG*	We will include any published CPGs which: include recommendations that relate to the assessment and investigation of hydration status, and any subsequent diagnosis and treatment of dehydration, after stroke. have been endorsed by a national and/or international organisation (e.g., governmental, charitable, professional practice) were published from January 2009 onwards. are available in English. We will exclude CPGs which: relate only to specific neuro(surgical) techniques for the treatment of stroke. provide recommendations for care outwith the acute setting (e.g., pre-hospital, rehabilitation units, and community). While consensus statements will not be included in appraisal and analysis, they may be of interest to support narrative discussion.
**R**	*Recommendation characteristics and “other” considerations*	Recommendations will be included if they provide explicit advice or direction regarding the assessment, diagnosis, treatment and management of hydration status in the acute setting. Information will be extracted regarding clinical and non-clinical recommendations; for example, a clinical recommendation may include prescription of intravenous fluids to correct dehydration, whereas a non-clinical recommendation may be to encourage/support patients to consume increased oral fluids. Informal text regarding hydration care will be extracted to elucidate the context within which recommendations are made.

**Table 2:** Characteristics of included international clinical practice guidelines.

Country	Organisation	Lead Author/Year	Title	Development Approach
Australia/ New Zealand	Stroke Foundation/ Australian Department of Health	Not stated (2026) [48]	Australian and New Zealand Living Clinical Guidelines for Stroke Management	Systematic search GRADE framework
Brazil	Brazilian Academy of Neurology	Oliveira-Filho (2012) [49]	Guidelines for acute ischemic stroke treatment—part I	Members from Brazilian stroke society participated in web-based discussion forum with pre-defined themes, followed by a formal onsite meeting OXFORD Classification (OCEBM)
Canada	Canadian Stroke Consortium, Canada’s national organization of stroke physicians	Heran (2022) [50]	Canadian Stroke Best Practice Recommendations: Acute Stroke Management, 7th Edition Practice Guidelines Update, 2022	Systematic search GRADE framework
China	Chinese Stroke Association	Liu (2023) [51]	Chinese Stroke Association guidelines for clinical management of ischaemic cerebrovascular diseases: 2023 update	Systematic search Chinese Stroke Association guideline grading system, modified AHA/ASA
India	Ministry of Health & Family Welfare– Govt. of India	Not stated (2019) [52]	Guidelines for Prevention and Management of Stroke	Not stated
Japan	Japan Stroke Society	Miyamoto (2022) [53]	Japan Stroke Society Guideline 2021 for the Treatment of Stroke	Systematic search The Japanese Stroke Society (JSS) guideline grading system, modified GRADE
Malaysia	Malaysian Society of Neurosciences/Academy of Medicine Malaysia	Not stated (2020) [54]	Clinical Practice Guidelines: Management of Ischaemic Stroke. Third Edition.	Systematic search Grading framework modified from the U.S/Canadian Preventative Services Task Force and the Guidelines for Clinical Practice Guideline
Pakistan	Pakistan Society of Neurology	Kamal (2010) [55]	Ischemic stroke care—official guidelines from the Pakistan society of Neurology	The primary author reviewed all the data on current stroke identification, management and secondary prevention as well as international guidelines. The manuscript was reviewed by several national neurology practitioners that encounter stroke patients
Qatar	Ministry of Public Health	Not stated (2020) [56]	National Clinical Guidelines. The diagnosis and management of stroke and transient ischaemic attack	Systematic search Modified American Speech Language Hearing Association Framework
Singapore	Ministry of Health	Venketasubramanian (2009) [47]	Stroke and Transient Ischaemic Attacks. Assessment, Investigation, Immediate Management, and Secondary Prevention.	Based on the SIGN Clinical Practice Guidelines on the Management of Patients with Stroke, which were reviewed and modified to meet local needs SIGN Evidence Framework
South Africa	South African Stroke Society (SASS) and the SASS Writing Committee	Bryer (2010) [57]	South African guideline for management of ischaemic stroke and transient ischaemic attack 2010: A guideline from the South African Stroke Society (SASS) and the SASS Writing Committee	At a meeting of the Stroke Guideline Writing Committee, authors nominated by consensus to write chapters which were reviewed at a national consensus meeting (150 delegates) and edited by the 1st author European Stroke Organisation definitions of levels of evidence
United Kingdom	Intercollegiate Stroke Working Party	Not stated (2023) [58]	National Clinical Guideline for Stroke for the UK and Ireland	Systematic search Not graded, strength either strong or conditional, accredited by NICE
USA	American Heart Association/American Stroke Association	Prabhakaran (2026) [59]	2026 Guideline for the Early Management of Patients With Acute Ischemic Stroke: A Guideline From the American Heart Association (AHA)/American Stroke Association	Systematic search AHA COR and LOE grades

AHA indicates American Heart Association; COR Class of Recommendation; GRADE Grading of Recommendations Assessment, Development and Evaluation; LOE Level of Evidence; OCEBM Oxford Centre for Evidence-Based Medicine; NICE National Institute for Health and Care Excellence; and SIGN Scottish Intercollegiate Guidelines Network.

**Table 3:** AGREE II appraisal of included guidelines.

GUIDELINE QUALITY ASSESSMENT AGREE II	Domain 1: Scope & Purpose	Domain 2: Stakeholder Involvement	* Domain 3: * Rigour of Development	Domain 4: Clarity of Presentation	Domain 5: Applicability	Domain 6: Editorial Independence	Domain 7: Overall Assessment
Clinical Practice Guidelines							
Australia, 2026 [48]	99	93	89	79	70	73	6
Brazil, 2012 [49]	33	26	17	63	0	0	1
Canada, 2023 [50]	96	93	84	68	61	98	6
China, 2023 [51]	68	8	36	35	0	21	1
India, 2019 [52]	33	28	2	38	0	2	0
Japan, 2021 [53]	69	43	67	63	13	83	4
Malaysia, 2020 [54]	94	47	80	75	64	0	4
Pakistan, 2010 [55]	32	1	7	18	9	0	0
Qatar, 2020 [56]	83	50	65	50	5	75	3
Singapore, 2009 [47]	82	76	44	35	18	2	2
South Africa, 2010 [57]	74	44	35	74	4	81	3
United Kingdom, 2023 [58]	100	97	89	71	66	88	6
USA, 2026 [59]	88	39	74	89	13	46	3
Median domain score (%)	82	44	65	63	13	46	
							
**AGREE II Score**	≥60% domain effectively addressed						
**High Quality**	Adequately address at least 3 domains, including domain 3*						
**Moderate Quality**	Adequately addresses 3 domains, except domain 3; Or adequately addresses 2 domains with a score of >50% in domain 3						
**Low Quality**	Guidelines not meeting the criteria for ‘high’ or ‘moderate’ quality						

## Data Availability

No new data were created or analysed in this study. Data sharing is not applicable to this article.

## Supplementary Materials

The following supporting information can be downloaded at: https://www.mdpi.com/article/10.3390/nu18111672/s1, Supplementary Materials S1: PRISMA Checklist; Supplementary Materials S2: Databases and search queries; Supplementary Materials S3: Clinical Practice Guideline Recommendations. References [47,48,49,50,51,52,53,54,55,56,57,58,59] are cited in the Supplementary Materials S3.

## Abbreviations

The following abbreviations are used in this manuscript:

AGREE	Appraisal of Guidelines for Research and Evaluation Tool
CPG	Clinical Practice Guidelines
CPGR	Clinical Practice Guideline Recommendation
DVT	Deep Vein Thrombosis
MeSH	Medical Subject Headings
PICAR	Population, Intervention, Comparator, Attributes of CPG and Recommendations
PRISMA	Preferred Reporting Items for Systematic Review and Meta-Analysis
U&E	Urea and Electrolytes
UK	United Kingdom
USA	United States of America
