# Supplementary material for: Hydration Care After Stroke: A Systematic Review of International Clinical Practice Guidelines
Source: Nutrients. 2026 May 23;18(11):1672. doi: 10.3390/nu18111672 (PMC13258263; doi:10.3390/nu18111672)
Supplement: Supplementary file 1 [file nutrients-18-01672-s001.zip › CMIL14 CPGSR - SuppMat S2 - Databases and search queries.pdf]

Hydration Guideline Review

Databases and Searches

Database: Ovid MEDLINE(R) and Epub Ahead of Print, In-Process, In-Data-Review & Other Non-Indexed Citations and Daily

----

1 exp Guideline/

2 Guideline.m_titl.

3 Guidelines.m_titl.

4 exp Practice Guideline/

5 Protocol.mp.

6 Clinical Management.mp.

7 exp Stroke/

8 Stroke.mp. [mp=title, abstract, original title, name of substance word, subject heading word, floating sub-heading word, keyword heading word, organism supplementary concept word, protocol supplementary concept word, rare disease supplementary concept word, unique identifier, synonyms]

9 Cerebrovascular Accident.mp. [mp=title, abstract, original title, name of substance word, subject heading word, floating sub-heading word, keyword heading word, organism supplementary concept word, protocol supplementary concept word, rare disease supplementary concept word, unique identifier, synonyms]

10 exp Dehydration/bl, cl, co, di, dg, en, ep, me, mo, nu, ph, pc, th, ur [Blood, Classification, Complications, Diagnosis, Diagnostic Imaging, Enzymology, Epidemiology, Metabolism, Mortality, Nursing, Physiology, Prevention & Control, Therapy, Urine]

11 Dehydration.mp. [mp=title, abstract, original title, name of substance word, subject heading word, floating sub-heading word, keyword heading word, organism supplementary concept word, protocol supplementary concept word, rare disease supplementary concept word, unique identifier, synonyms]

12 exp Fluid Therapy/

13 Fluid Therapy.mp. [mp=title, abstract, original title, name of substance word, subject heading word, floating sub-heading word, keyword heading word, organism supplementary concept word, protocol supplementary concept word, rare disease supplementary concept word, unique identifier, synonyms]

14 Rehydration.mp. [mp=title, abstract, original title, name of substance word, subject heading word, floating sub-heading word, keyword heading word, organism supplementary concept word,

protocol supplementary concept word, rare disease supplementary concept word, unique identifier, synonyms]

15 Hydration.mp. [mp=title, abstract, original title, name of substance word, subject heading word, floating sub-heading word, keyword heading word, organism supplementary concept word, protocol supplementary concept word, rare disease supplementary concept word, unique identifier, synonyms]

16 Hydration Therapy.mp. [mp=title, abstract, original title, name of substance word, subject heading word, floating sub-heading word, keyword heading word, organism supplementary concept word, protocol supplementary concept word, rare disease supplementary concept word, unique identifier, synonyms]

17 Fluid.mp. [mp=title, abstract, original title, name of substance word, subject heading word, floating sub-heading word, keyword heading word, organism supplementary concept word, protocol supplementary concept word, rare disease supplementary concept word, unique identifier, synonyms]

18 1 or 2 or 3 or 4 or 5 or 6

19 7 or 8 or 9

20 10 or 11 or 12 or 13 or 14 or 15 or 16 or 17

21 18 and 19 and 20

22 limit 21 to yr="2009 -Current"

Database: HMIC Health Management Information Consortium

----

1 exp Clinical guidelines/

2 exp Guidelines/

3 Guideline.m_titl.

4 Guidelines.m_titl.

5 exp Stroke/

6 Stroke.mp.

7 exp Fluid balance care/

8 exp Fluid therapy/

9 Dehydration.mp. [mp=title, other title, abstract, heading words]

10 Hydration.mp. [mp=title, other title, abstract, heading words]

11 1 or 2 or 3 or 4

12 5 or 6 or 7 or 8 or 9 or 10

13 11 and 12

Database: HMIC Health Management Information Consortium

----

1 exp Guideline/

2 Guideline.m_titl.

3 Guidelines.m_titl.

4 exp Practice Guideline/

5 Protocol.mp.

6 Clinical Management.mp.

7 exp Stroke/

8 Stroke.mp. [mp=title, abstract, heading word, drug trade name, original title, device manufacturer, drug manufacturer, device trade name, keyword, floating subheading word, candidate term word]

9 Cerebrovascular Accident.mp. [mp=title, abstract, heading word, drug trade name, original title, device manufacturer, drug manufacturer, device trade name, keyword, floating subheading word, candidate term word]

10 [exp Dehydration/bl, cl, co, di, dg, en, ep, me, mo, nu, ph, pc, th, ur [Blood, Classification, Complications, Diagnosis, Diagnostic Imaging, Enzymology, Epidemiology, Metabolism, Mortality, Nursing, Physiology, Prevention & Control, Therapy, Urine]]

11 Dehydration.mp. [mp=title, abstract, heading word, drug trade name, original title, device manufacturer, drug manufacturer, device trade name, keyword, floating subheading word, candidate term word]

12 exp Fluid Therapy/

13 Fluid Therapy.mp. [mp=title, abstract, heading word, drug trade name, original title, device manufacturer, drug manufacturer, device trade name, keyword, floating subheading word, candidate term word]

14 Rehydration.mp. [mp=title, abstract, heading word, drug trade name, original title, device manufacturer, drug manufacturer, device trade name, keyword, floating subheading word, candidate term word]

15 Hydration.mp. [mp=title, abstract, heading word, drug trade name, original title, device manufacturer, drug manufacturer, device trade name, keyword, floating subheading word, candidate term word]

16 Hydration Therapy.mp. [mp=title, abstract, heading word, drug trade name, original title, device manufacturer, drug manufacturer, device trade name, keyword, floating subheading word, candidate term word]

17 Fluid.mp. [mp=title, abstract, heading word, drug trade name, original title, device manufacturer, drug manufacturer, device trade name, keyword, floating subheading word, candidate term word]

18 1 or 2 or 3 or 4 or 5 or 6

19 7 or 8 or 9 or 10 or 11 or 12 or 13 or 14 or 15 or 16 or 17

20 18 and 19

21 limit 20 to yr="2009"

Database: CINAHL (EBSCO)

----

1 MH Practice Guidelines+

2 TI Guidelines

3 MH Stroke+

4 SU Stroke

5 TX Cerebrovascular Accident

6 MH Dehydration+

7 MH Fluid Therapy+

8 MH Hydration Status+

9 1 OR 2

10 3 OR 4 OR 5

11 6 OR 7 OR 8

12 9 AND 10 AND 11

13 Limiters published 2009
