# Supplementary material for: Hydration Care After Stroke: A Systematic Review of International Clinical Practice Guidelines
Source: Nutrients. 2026 May 23;18(11):1672. doi: 10.3390/nu18111672 (PMC13258263; doi:10.3390/nu18111672)
Supplement: Supplementary file 1 [file nutrients-18-01672-s001.zip › supplementary S3.pdf]

NO	COUNTRY	ORGANISATION	LEAD AUTHOR	YEAR	REF	QUALITY	RECOMMENDATION N ALL	N CPG	Recommendations relating to the assessment/ investigation, diagnosis, management/treatment of hydration status after stroke	GRADE/ CLASS	LOE
1	AUSTRALIA/ NEW ZEALAND	Stroke Foundation	Not stated	2026	58	High	1	1	**Chapter 3 Acute medical and surgical management: Dysphagia: Practice Statement: Consensus-based recommendations; Until a safe swallowing method is established for oral intake, patients with dysphagia should have their** nutrition and **hydration assessed and managed with early consideration of alternative non-oral routes.**	CB	Not stated
							2	2	**Chapter 3 Acute medical and surgical management: Dysphagia: Practice Statement: Consensus-based recommendations; Patients with dysphagia on texture-modified** diets and/or **fluids should have their intake and tolerance to the modified diet monitored regularly due to the increased risk of** malnutrition and **dehydration.**	CB	Not stated
							3	3	**Chapter 3 Acute medical and surgical management: Dysphagia: Practice Statement: Consensus-based recommendations; Patients with persistent** weight loss, **dehydration** and/or recurrent chest infections **should be urgently reviewed.**	CB	Not stated
							4	4	**Chapter 6 Managing complications: Nutrition and hydration: Early hydration; All patients with stroke should have their hydration status assessed, monitored, and managed throughout their hospital admission.**	Strong	Low
							5	5	**Chapter 6 Managing complications: Nutrition and hydration: Early hydration; Where fluid support is required, crystalloid solution should be used in preference to colloid solutions as the first option to treat or prevent dehydration.**	Strong	Low
2	BRAZIL	Brazilian Stroke Society/ Brazilian Academy of Neurology	Oliveira-Filho, J.	2012	50	Low	6	1	**Pre-hospital care and transportation: 5; To adopt, in all pre-hospital transportation: monitorization of** oxygen saturation, and in all who have saturation below 95% to apply oxygen supplementation; **clinical evaluation of hydration and, in case of dehydration, to apply intravenous hydration with 0.9% normal saline;** finger stick testing for blood glucose, and to dispense glucose containing fluids only in case of detected hypoglycemia; and not to treat systemic hypertension routinely.	B	1
							7	2	**Stroke in the emergency department: 10; The patient with acute stroke should be adequately hydrated with isotonic saline solution (e.g. 0.9% saline). The volume administered intravenously (around 100 mL/hour) should be considered individually, based on the baseline hydration and possible comorbidities that limit water supply.**	C	4
3	CANADA	Canadian Stroke Consortium/ Canada's national organization of stroke physicians	Heran, M.	2022	55	High	8	1	**4.3.D Emergency department evaluation and management of patients with TIA and acute stroke: Urethral catheters; … Fluid status** and urinary retention **should be included as part of routine monitoring of vital sign assessments.**	Strong	Moderate
							9	2	**9.2.1 Inpatient prevention and management of complications: Venous Thromboembolism Prophylaxis;** All patients with stroke should be assessed for their risk of developing venous thromboembolism... **Patients at high risk include** those who are unable to move one or both lower limbs ;those who are unable to mobilize independently; those with a previous history of venous thromboembolism; **those who are dehydrated**; and those with comorbidities such as active or suspected malignancy.	Strong	Moderate
							10	3	**9.6.1 Inpatient prevention and management of complications: Nutrition and Dysphagia; Patients should be screened for swallowing impairment before any oral intake, including** medications, food, and **liquid, by an appropriately trained professional using a valid screening tool.**	Strong	Moderate
							11	4	**9.6.2 Inpatient prevention and management of complications: Nutrition and Dysphagia; The** swallowing, nutritional and **hydration status of patients with stroke should be screened as early as possible, ideally within 24 hours of admission, using validated screening tools.**	Strong	Moderate
							12	5	**9.6.3 Inpatient prevention and management of complications: Nutrition and Dysphagia; Abnormal results from the initial or ongoing swallowing screens should trigger a prompt referral to a speech-language pathologist, occupational therapist, dietitian, and/or other trained dysphagia clinicians for more detailed assessment and management** of swallowing, feeding, nutritional, and **hydration status.**	Strong	Moderate
							13	6	**9.6.4 Inpatient prevention and management of complications: Nutrition and Dysphagia; For patients who cannot safely swallow or meet their** nutrient and **fluid needs orally, enteral nutrition (e.g., nasogastric tube feeding) should be considered** in consultation with the patient, family, or substitute decision-maker, and the interdisciplinary team **as early as possible after admission, usually within the first three days of admission.**	Strong	Moderate

4	CHINA	Chinese Stroke Association	Liu L.	2023	56	Low	14	1	**6.1.4 General supportive care: Dysphagia;** **Dysphagia screening before the patient begins** eating, **drinking** or receiving oral medications is reasonable, which may help to identify patients at high risk of aspiration.	2a	C
							15	2	**6.1.6 General supportive care: Deep vein thrombosis prophylaxis;** **For patients who had a stroke** with limited mobility and no contraindications, in addition to **conventional treatment** (aspirin and **fluid therapy**), intermittent pneumatic compression is recommended to reduce the risk of DVT.	1	B
5	INDIA	Ministry of Health & Family Welfare	Not stated	2019	51	Low	16	1	**5.3.1 Management of established acute stroke: History, physical examination and common investigations;** **History should follow the usual routine. Special attention should be paid to** time of onset of symptoms, recent stroke, myocardial infarction, seizure, trauma, surgery, bleeding, pregnancy, vegetarianism and use of anticoagulation / insulin / antihypertensive, history of modifiable risk factors: hypertension, diabetes, smoking, non-smoking tobacco use, heart disease, hyperlipidaemia, migraine, and history of headache or vomiting, recent childbirth, **risk of dehydration.**	Not stated	Not stated
							17	2	**5.7.F.7 Management of established acute stroke: Cerebral oedema and increased intracranial pressure (ICP);** **Strict intake-output chart must be maintained to avoid dehydration.**	Not stated	Not stated
							18	3	**5.7.G.2.5 Management of established acute stroke: General early supportive care: Swallowing;** **Patients with altered sensorium should be given only intravenous fluids (Dextrose saline or normal saline)** for at least 2-3 days, followed by nasogastric tube feeding.	Not stated	Not stated
							19	4	**5.7.G.6.1.4 Management of established acute stroke: General early supportive care: Deep venous thrombosis: Prophylaxis against DVT;** Early mobilization and **optimal hydration should be maintained for all acute stroke patients.**	Not stated	Not stated
6	JAPAN	Japan Stroke Society	Miyamoto, S.	2021	54	High	20	1	**2.5.2 Acute management of stroke in general: General management - Nutrition;** **In patients with consciousness disorder, dysphagia, or unstable neurological or systemic status, start intravenous fluid replacement with avoidance of oral intake is recommended.**	A	Moderate
7	MALAYSIA	Malaysian Society of Neurosciences/ Ministry of Health Malaysia	Not stated	2020	52	High	21	1	**6.2.1.3 Investigations: Haematological investigations;** **Urea & electrolytes - Hydration status, excludes electrolyte imbalances**	Not stated	Not stated
							22	2	**8.1.11 Acute general management: Deep vein thrombosis;** **For immobile stroke patients** without contraindications, intermittent pneumatic compression (IPC) in addition to **routine care** (aspirin and **hydration**) is recommended over routine care alone to reduce the risk of deep vein thrombosis (DVT).	A	1
8	PAKISTAN	Pakistan Society of Neurology	Kamal, A.	2010	48	Low	23	1	**2.7 Management issues and recommendations: Nutrition and hydration;** **Patients should receive isotonic hydration and free fluids should be avoided.** Nutritional supplementation is not necessary. However an evaluation for aspiration is needed prior to initiation of diet and the diet should be modified accordingly.	Not stated	Not stated
9	QATAR	Ministry of Public Health	Not stated	2020	53	High	24	1	**8.2.3 Care in the specialised stroke unit: Swallowing assessment;** **Patients may benefit from intravenous normal saline to maintain hydration.**	Not stated	Not stated
							25	2	**8.2.4 Care in the specialised stroke unit: Swallowing assessment;** **Do not use hypotonic fluids in patients with acute stroke.**	Not stated	Not stated
							26	3	**8.3.8 Care in the specialised stroke unit: Assess for and manage complications;** **Observe patients for the development of common early complications including… Dehydration and malnutrition.**	Not stated	Not stated
10	SINGAPORE	Ministry of Health	Venketasubramanian, N	2009	47	Low	27	1	**3.5. Immediate management following acute stroke: Medical management: Venous thromboembolism;** **Good hydration** and early mobilisation **is recommended for all stroke patients** to reduce deep venous thrombosis and pulmonary embolism.	D	2

11	SOUTH AFRICA	South African Stroke Society	Bryer, A.	2011	49	Moderate	28	1	**8.4.1 Management of acute ischaemic stroke: Treatment: General supportive treatment to maintain homeostasis and treatment of complications:** **Regular monitoring of fluid balance and electrolytes is recommended in patients with severe stroke or swallowing problems**	4	GCP
							29	2	**8.4.1 Management of acute ischaemic stroke: Treatment: General supportive treatment to maintain homeostasis and treatment of complications:** **Normal saline (0.9%) is recommended for fluid replacement during the first 24 hours after stroke.**	4	GCP
							30	3	**10 Management of common systemic complications of acute ischaemic stroke: Prevention of DVT and PE;** **Early rehydration is recommended to reduce the incidence of venous thromboembolism.**	4	GCP
12	UK	Intercollegiate Stroke Working Party	Not stated	2023	57	High	31	1	**2.4.D Organisation of stroke services: Organisation of inpatient stroke services: Recommendations;** **Acute stroke services should have management protocols for** the admission pathway including links with the ambulance service, emergency stroke treatments, acute imaging, neurological and physiological monitoring, swallowing assessment, **hydration and nutrition**, vascular surgical referrals, rehabilitation, end-of-life (palliative) care, secondary prevention, the prevention and management of complications, communication with people with stroke and their family/carers and discharge planning.	Not stated	Not stated
							32	2	**3.10.B Acute care: Acute stroke care: Recommendations;** **Patients with acute stroke should have their clinical status monitored closely, including:** level of consciousness; blood glucose; blood pressure; oxygen saturation; **hydration and nutrition**; temperature; cardiac rhythm and rate.	Not stated	Not stated
							33	3	**3.10.D Acute care: Acute stroke care: Recommendations;** **Patients with acute stroke should have their hydration assessed using a standardised approach within four hours of arrival at hospital, and should be reviewed regularly and managed so that normal hydration is maintained.**	Not stated	Not stated
							34	4	**3.10.F Acute care: Acute stroke care: Recommendations;** **Until a safe swallowing method is established, patients with dysphagia after acute stroke should: be immediately considered for alternative fluids;** have a comprehensive specialist assessment of their swallowing; be considered for nasogastric tube feeding within 24 hours; be referred to a dietitian for specialist nutritional assessment, advice and monitoring; **receive adequate hydration,** nutrition and medication **by alternative means**; be referred to a pharmacist to review the formulation and administration of medication.	Not stated	Not stated
13	USA	American Heart/Stroke Associations	Prabhakaran, S.	2026	59	High	35	1	**5.2.1 Dysphagia:** **In patients with AIS, performing a bedside swallow screening prior to initiation of liquid** or food **intake is recommended to screen for patients at increased risk for aspiration.**	1	C-EO
